# Measuring inequality in quality of life: Why the EQ-5D may underestimate it

**DOI:** 10.1007/s11136-025-04087-7

**Published:** 2025-11-11

**Authors:** Jan Abel Olsen, Gang Chen, Admassu Lamu

**Affiliations:** 1 Department of Community Medicine, Faculty of Health Sciences, UiT - the Arctic University of Norway, 9019 Tromsø, Norway; 2 Cancer Health Services Research, The University of Melbourne, Melbourne, Australia; 3 Department of Health and Social Sciences, NORCE Norwegian Research Centre, Bergen, Norway

**Keywords:** EQ-5D-5L, EQ-VAS, Health inequality, Education-health gradient

## Abstract

**Objective:**

The EQ-5D is increasingly being used in studies of health inequalities, providing further evidence of a social gradient in health; i.e. consistent positive associations between a socioeconomic indicator and health. However, the steepness in the social gradients in HRQoL differs depending on which of the two EQ-5D measures is used; whether based on respondents’ EQ-5D-5L *descriptions* or by their *direct valuation* in EQ-VAS. This study aims to provide new knowledge as to why the two HRQoL measures suggest different degrees of health inequalities.

**Methods:**

Based on two large unique data sets (Tromsø Study Wave 7, N = 21,083; MIC study, N = 8022), cross-sectional analyses were conducted. We identified the most prevalent EQ-5D-5L profiles. Within each of ten EQ-5D-5L profile groups, we examine response heterogeneities in EQ-VAS scores using linear regressions, as explained by respondents’ level of educational attainment, controlling for age and sex.

**Results:**

We showed significantly increasing EQ-VAS scores along with educational attainments. For instance, in the most prevalent health state (11121), a consistent education-health gradient was observed: compared to individuals with primary education, the EQ-VAS was 1.7 higher among those with secondary education; 2.6 higher among individuals with short tertiary education; and, 3.7 higher among individuals with long tertiary education.

**Conclusions:**

This paper provides new insights into the use of EQ-5D in health inequality studies by suggesting an additional underlying education gradient in HRQoL than what is revealed through the EQ-5D-5L values. Broader psychosocial domains and aspects of adaptation should be considered when monitoring health inequalities.

**Supplementary Information:**

The online version contains supplementary material available at 10.1007/s11136-025-04087-7.

## Abstract (plain language summary)

The existence of social inequalities in life expectancies is widely documented. With more health survey data becoming available, there is now an increasing interest in studying social inequalities, or the social gradient, in quality of life. The EQ-5D is the most widely used health utility instrument, providing two different quality of life measures: one based on respondents’ EQ-5D-5L description and another based on their direct valuation on a visual analogue scale (EQ-VAS).Recent studies have shown the magnitude of health inequalities, or the steepness in the social gradients, to differ depending on which of the two measures is used. This study aims to provide new knowledge as to why this is so.Based on two large data sets, we identified the most prevalent health states, described by EQ-5D-5L profiles. We showed that individuals who have described their health state identically still differ in how they value their health on the VAS: individuals with better educational achievement generally report higher VAS-scores.This paper provides new insights into the use of EQ-5D in health inequality studies by showing an additional underlying education-health gradient than what is revealed through the EQ-5D-5L values. In short, the EQ-5D-5L appears to underestimate the inequality in quality of life.

**Supplementary Information:**

The online version contains supplementary material available at 10.1007/s11136-025-04087-7.

## Introduction

The EQ-5D is increasingly being used in population health surveys, providing population norms across sex- and age-groups in several countries. Based on such data, health inequalities – or the *social gradient in health*, defined as the increased health associated with each step up a socioeconomic ladder – have been investigated, showing consistent positive associations between the level of educational attainment as a proxy for socio-economic position (SEP), and the EQ-5D-5L using level sum scores or country-specific value sets, as well as by use of the EQ-VAS [1–3]. Furthermore, the EQ-5D has started to play an important role in evaluating the policy impact of health reforms that aim to tackle health inequalities [4].

The EQ-5D provides two fundamentally different measures of how population sub-groups differ in how they value their own health-related quality of life (HRQoL); either based on their EQ-5D-5L *description*, with corresponding index values from country-specific preference-based value sets, or by their *direct valuation* using EQ-VAS. When comparing the social gradients in HRQoL across these two measures, a recent Norwegian study showed the social gradients in HRQoL – split by gender – were twice as steep when using EQ-VAS scores as compared to EQ-5D-5L values [5]. The discrepancies between the highest and lowest education levels in the mean EQ-VAS score (when normalized to [0–1] scale) were 0.078 for women and 0.056 for men. When using EQ-5D-5L values, the corresponding discrepancies were 0.039 and 0.029, respectively. Thus, we observe a much stronger social gradient in how individuals *value* their health directly by the use of EQ-VAS, as compared to how they *describe* their health.

There could be two potential reasons why EQ-VAS would capture more inequality than EQ-5D-5L. First, *elements of adaptation* [6] suggest that individuals with low socioeconomic position may have lower expectations and thereby underreport their health problems on a structured health state classification system. Second, when responding to the EQ-VAS, individuals may consider broader *psychosocial health domains* beyond those covered by the EQ-5D-5L classification, and individuals with low socioeconomic position suffer more psychosocial problems [7].

The choice between EQ-5D values *versus* EQ-VAS scores would have important implications for health inequality monitoring and policy making. To the extent that systematic socioeconomic disparities in EQ-VAS are observed within identical EQ-5D-5L profiles, this is indicative that EQ-5D-5L values *underestimate* the inequality, i.e., may not capture the full picture of true differences in health between SEP groups. This paper provides new insights into the use of EQ-5D in studies of health inequality by suggesting that there is an additional underlying educational gradient in HRQoL beyond what is revealed through the EQ-5D-5L values. Based on two comprehensive data sets (Tromsø Study, N = 21,083; MIC study, N = 8022), this paper aims to investigate the potential under-estimation of education-health gradients when using EQ-5D-5L values as a proxy for health. More specifically, we identify the most prevalent EQ-5D-5L profiles and examine response heterogeneities in EQ-VAS scores *within* each EQ-5D-5L profile group, as explained by respondents’ level of educational attainment, controlling for age and sex. Finally, we discuss reasons why the EQ-VAS generally shows steeper education-health gradients than EQ-5D-5L.

## Methods

### Data

Our main data set is the latest wave of an ongoing population-based health study in the largest city in Northern Norway; The Tromsø Study, Wave 7, conducted in 2015/16 (N = 21,083, aged 40–93 years) [8]. The study population is considered representative of the Norwegian adult population, however, slightly overrepresented by people with a university degree. In the current study, we excluded 761 (3.6%) respondents aged 80 and above, due to the low response rate in this age group. Further, due to the much shorter educational attainment among the elderly, there was hardly any diversity along this key variable. The study was approved by the Regional Committee for Medical and Health Research Ethics (ID, 2016/607). All participants gave written informed consent before admission.

In addition, we use data from the largest international survey conducted to compare generic preference-based measures; the Multi-Instrument Comparison (MIC) project, with a total of 8022 participants from six countries (Australia, Canada, Germany, Norway, the UK, and the US). This data set includes targeted patient groups with prevalent chronic conditions, as well as a representative adult population group, referred to as the ‘healthy public’ [9, 10]. In the current study, we excluded two age groups: i) respondents who were under 25 (N = 467; 5.9%), i.e. the age where most students would have completed their education, and ii) those aged 80 and above (N = 102; 1.3%).

### Variables

HRQoL was measured by the EQ-5D-5L descriptive system, as well as the visual analogue scale EQ-VAS (see www.euroqol.org, [11]. The source for EQ-5D-5L index values is an aggregate value set based on the means of ten Western countries’ preference pattern (MN-WePP): Canada, Denmark, England, France, Germany, Ireland, The Netherlands, Portugal, Spain, USA [12]. This value set is anchored on a [0 (death) to 1 (full health)] scale but allows for negative values for health states considered worse than being dead.

The EQ-VAS score is based on respondents’ direct valuations of their overall health on a visual analogue scale that ranges from 0 (*worst imaginable health*) to 100 (*best imaginable health*). In the MIC study, the scale used had different framing and wording than the official EQ-VAS, i.e. it was anchored between zero (death) and 100 ‘Excellent Health – Physical, mental, emotional and social wellbeing are all at their best’. The different framing and wording could bias the result. However, our aim was *not* to compare the magnitude of VAS differences across the two data sources. Rather, we included the MIC data to seek supportive evidence that subjects who report identical EQ-5D-5L profiles will report differently on an alternative HRQoL measure, in this case, a differently framed VAS, as explained by their educational attainment. Interestingly, if such a pattern were also observed on differently framed HRQoL measures, it might provide further support for the existence of report heterogeneity in HRQoL by level of education.

Educational attainment is the most widely used indicator of SEP in this literature, despite its potential limitations in terms of cohort effects and reverse causality. In the Tromsø Study, it is reported along four levels: primary (including lower secondary); secondary (including vocational); tertiary low (less than 4 years of university study); and, tertiary high (4 years or more of university study). In the MIC study, it was reported along three levels: high school; diploma/certificate; and university. However, we acknowledge potential comparability issues on the education variable across the six countries.

### Statistical analysis

Since EQ-VAS is a global evaluation of health that would cover wider aspects of HRQoL than those described by the five EQ-5D dimensions, we expect some reasonable discrepancies in responses to the two measures. However, for reasons of exclusion due to suspected inconsistent responses between EQ-VAS and EQ-5D-5L, we considered a conservative cut-off point between the two: EQ-VAS < 0.50 (≈3SD) for the 11111 health profile, and < 0.30 for the rest on a 0–1 scale (See Appendix Table A1).

**Table 1:** Respondent characteristics

	Tromsø Study			MIC-study		
	N	%	Mean VAS (SD)	N	%	Mean VAS SD)
*Gender*						
Male	9661	47.5	76.6 (15.4)	3566	48.4	67.2 (21.7)
Female	10,661	52.5	76.2 (16.9)	3798	51.6	67.7 (21.6)
*Age groups*^*a*^						
25–39 years	n.a	n.a	n.a	1423	19.3	69.6 (20.5)
40–59 years	12,467	61.4	76.7 (15.9)	3259	44.3	65.0 (22.6)
60–79 years	7855	38.7	75.8 (16.5)	2682	36.4	69.3 (20.7)
*Educational attainment*^*b*^						
Primary	4408	22.0	72.7 (17.8)	2257	30.7	65.9 (22.2)
Secondary	5596	28.0	75.5 (16.2)	3014	40.9	66.6 (21.7)
Tertiary Low	3930	19.6	77.0 (15.6)	2093	28.4	70.3 (20.7)
Tertiary High	6093	30.4	79.4 (14.4)			
*Chronic conditions*						
Not diagnosed				1575	21.4	84.0 (8.60)
Diagnosed				5789	78.6	62.9 (21.9)
*Country*						
Norway	20,322	100		1057	14.4	70.4 (20.7)
Australia				1277	17.3	63.4 (22.0)
Canada				1221	16.6	69.5 (19.6)
Germany				1194	16.2	65.1 (22.1)
UK				1241	16.9	64.2 (22.8)
US				1374	18.7	69.3 (21.7)

*MIC*: Multi Instrument Comparison study; *EQ-VAS*: EuroQoL Visual Analogue Scale; *SD*: Standard Deviation; *n.a*.: not available. ^a^Minimum age for inclusion in the Tromsø Study was 40; we chose minimum age of 25 for MIC data to account for university education. ^b^Educational attainment in the MIC survey was split into three categories (High school, Diploma/certificate, University)

To allow for sufficient numbers of cases that had reported an identical EQ-5D-5L profile (described using 5-digit numbers), we sorted the 3125 unique EQ-5D-5L profiles in order of their prevalence, preferably covering a spectrum of severities. The seven most frequent profiles included 77.3% of the total Tromsø data. The distributions of EQ-VAS within each of these profiles are presented in Appendix Figure A1. However, only one profile had a moderate/severe level (11131). Hence, we chose to include the three most frequent profiles with a level 3 or higher included (11132; 11123; 21231). We then had 10 different profiles, which together represent 80.5% of the total sample. The minimum sample size required to detect Cohen’s small effect size [13], assuming five predictors in the model (three education dummies, age and sex), with a significance level of 5%, and a power of 90% is 189. However, the next most prevalent profile with a moderate level has fewer cases.

**Fig. 1:**
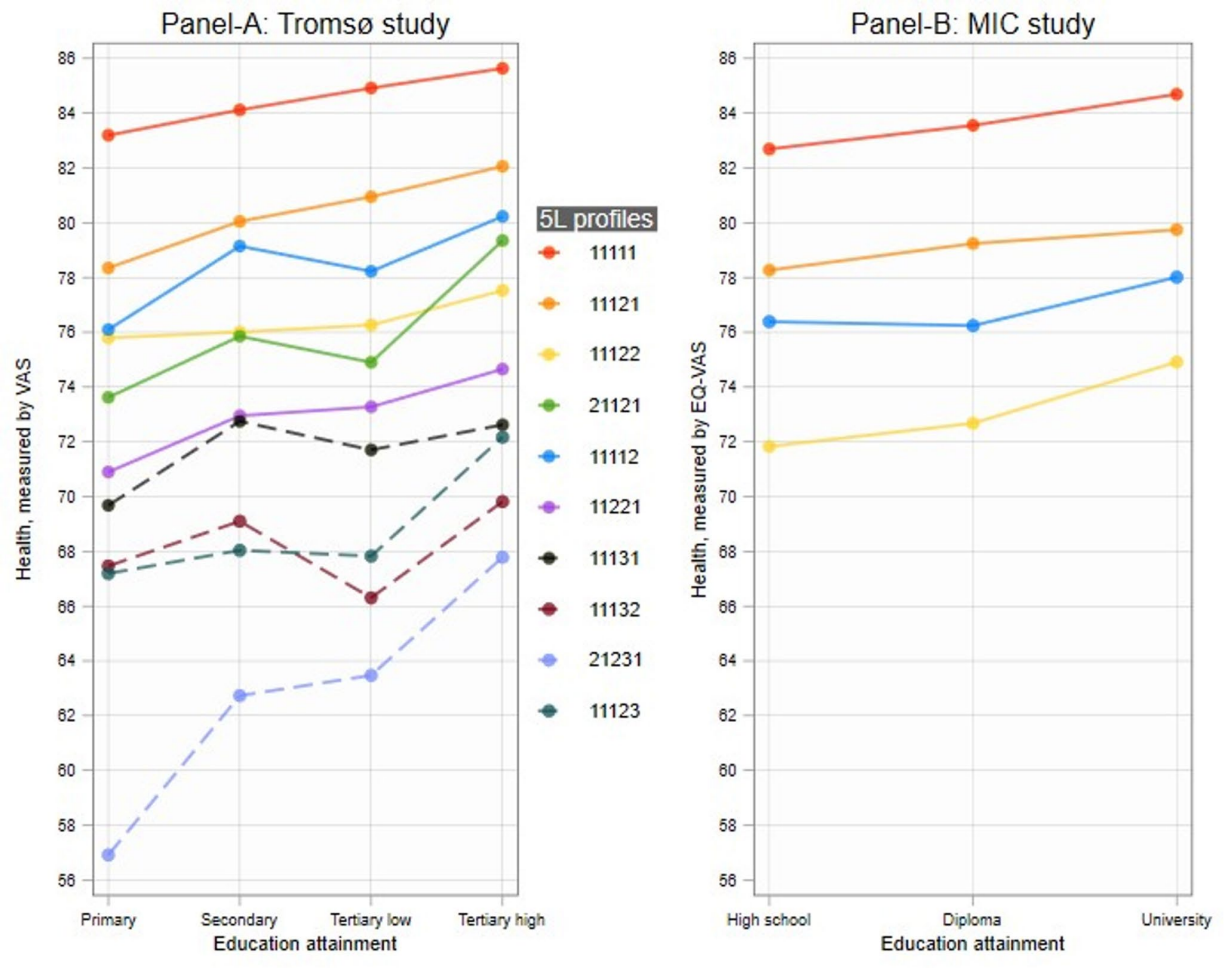
Mean EQ-VAS by education within each EQ-5D-5L profile, adjusted for age and sex. EQ-*VAS*: EuroQoL Visual Analogue Scale. Tertiary low is < 4 years of university education; Tertiary high is ≥ 4 years of university education. Panel-A depicts mean EQ-VAS by education for ten selected EQ-5D-5L profiles in the Tromsø study (broken lines represent moderate-level profiles), and Panel-B for the MIC study using a different framing of VAS

For higher statistical power and precision, we also estimated the minimum sample size required to detect a small effect size in the MIC data. With ten predictors in the model (including two education dummies, five control dummies, chronic condition, age and sex), a sample of 238 cases is required to detect a small effect size, which gave four profiles that we included.

The selected EQ-5D-5L profiles are detailed in Appendix Table A2. Within each profile considered, we report the distribution of EQ-VAS scores and present the mean scores by education level. We use a graph to illustrate the distribution of mean EQ-VAS by education.

**Table 2:** Linear regressions of EQ-VAS score on education attainment within selected EQ-5D-5L profiles: The Tromsø Study (40–79 years)

	Most prevalent 5L profiles						Moderate level profiles			
Variables	11111	11121	11122	21121	11112	11221	11131	11132	21231	11123
*Education*										
Secondary	0.940**	1.710***	0.213	2.227	3.026*	2.050	3.065**	1.632	5.798**	0.848
	(0.473)	(0.461)	(1.021)	(1.455)	(1.750)	(1.722)	(1.418)	(2.281)	(2.334)	(2.945)
Tertiary low	1.750***	2.614***	0.471	1.278	2.112	2.374	2.022	−1.164	6.536*	0.635
	(0.496)	(0.497)	(1.097)	(1.662)	(1.831)	(2.184)	(1.824)	(2.596)	(3.539)	(3.124)
Tertiary high	2.478***	3.731***	1.738*	5.715***	4.102**	3.750*	2.937*	2.350	10.837***	5.015*
	(0.456)	(0.464)	(1.016)	(1.514)	(1.673)	(1.972)	(1.575)	(2.598)	(2.936)	(2.994)
*Sex*										
Female	2.729***	1.951***	1.292*	0.884	1.495	−1.366	0.815	0.158	0.178	0.162
	(0.297)	(0.308)	(0.684)	(1.078)	(1.096)	(1.394)	(1.096)	(1.761)	(2.162)	(1.889)
Constant	80.512***	78.262***	77.225***	77.139***	71.613***	73.885***	75.736***	70.964***	61.670***	63.953***
	(1.016)	(1.038)	(2.333)	(3.614)	(3.957)	(4.427)	(3.667)	(5.917)	(6.267)	(6.728)
Observations	5 440	5 695	1 417	631	556	407	653	230	195	189
R-squared	0.023	0.022	0.008	0.032	0.014	0.014	0.020	0.013	0.084	0.024
Cohen's *f*	0.153	0.150	0.090	0.182	0.119	0.119	0.143	0.115	0.303	0.157

*EQ-VAS*: EuroQol Visual Analogue Scale; Standard errors in parentheses, *** *p* < 0.01, ** *p* < 0.05, * *p* < 0.1. All models controlled for age

For each EQ-5D-5L profile and each of the two data sets, we run separate linear regression analyses on EQ-VAS score, with educational attainment dummies as key explanatory variables of interest. All regressions control for age and sex. In the MIC data, as chronic disease samples were purposely recruited, we further controlled for chronic condition dummies and country dummies. To contextualise results in terms of policy relevance, we reported Cohen’s *f* statistic, which measures effect sizes – the strength of the relationship between variables [13]. In line with guidelines from literature, *f* ≥ *0.05, f* ≥ *0.10, f* ≥ *0.20, f* ≥ *0.30, f* ≥ *0.40* represent *very small, small, medium, large, and very large* effect sizes, respectively [14].

We checked the robustness of our results by using a robust regression estimator, i.e. a maximum likelihood-type estimator (M-estimator), which is less influenced by potential outliers in the data [15]. We also replicated our analyses using a semi-parametric fractional logit regression model, which uses a quasi-maximum likelihood estimator that does not require distributional assumptions between the dependent and independent variables [16, 17]. The analyses from both the M-estimators and the fractional logit are quite similar to those of the linear regression. Thus, we focus on linear regression analyses based on the principle of parsimony, leaving results from the M-estimators and fractional logit in the Appendix.

We further investigate alternative specifications, including interaction terms, to check whether the impact of education on health varies by the covariates included and other potential confounders. We found no evidence of strong interactions. Nevertheless, we assessed the education-gradient in EQ-VAS by sex to explore variation in inequality between males and females.

After scrutinising the data, we found a few missing values for key variables: EQ-5D-5L (< 4%), EQ-VAS and education variables (< 2%). When missing data is small (< 5%), the potential impact of missingness is likely trivial [18]. However, to preserve the full sample, we applied the Full Information Maximum Likelihood (FIML) method in the linear regression model. The FIML approach utilises observed information from all variables in the model to impute missing data, which yields unbiased parameter estimates when missing data are at random. No missing data were observed in the MIC survey. Thus, we conducted a complete case analysis for the MIC survey.

## Results

Respondent characteristics are reported in Table 1. The description of the most frequent EQ-5D-5L profiles is reported in Table A2. Note the concentration; out of potentially 3125 unique health profiles, the six most prevalent profiles cover 75% of this adult population sample of the Tromsø Study. In the MIC study, which includes patient populations with common chronic conditions, the four most prevalent profiles cover more than 46%.

Regression results based on Tromsø data are presented in Table 2, with the predictive margins illustrated by the gradients in Fig. 1, Panel A. The two most prevalent profiles consistently show significant education gradients, i.e. EQ-VAS score increases along each education level. In the profile (11121), compared to individuals with primary education, the EQ-VAS was 1.7 higher among those with secondary education, 2.6 higher among individuals with short tertiary education, and 3.7 higher among individuals with long tertiary education. These results are higher than the lowest minimum important change of 0.96 reported in the literature [19]. The general pattern is the same for the less prevalent profiles, except for slight dips at the third education level (Tertiary low), as compared to the second education level, in five profiles. It is hard to tell whether these dips reflect cultural differences or sampling artefacts. Still, in nine out of ten health state profiles, the EQ-VAS was highest among individuals with the highest education level and lowest among individuals with the lowest education level. No significant differences were observed in the one remaining profile (11132), which had an admittedly low number of observations (N = 230).

Regression results from MIC data are reported in Appendix Table A3, together with its predictive margins by education in Panel B of Fig. 1. The same general pattern is observed, with all profiles showing consistent education gradients in the VAS. Results from fractional logit (Appendix Table A4) and robust regression analyses based on M-estimators (Appendix Fig. A2) confirm these findings.

While our regressions show consistent differences, another matter is whether the effect sizes are clinically meaningful, or represent a minimally important difference (MID). With reference to results from a recent systematic review, it has been suggested that the distribution-based MID estimates for EQ-VAS range from 0.96 to 16.6 using distribution-based approaches [19]. Thus, while our effect sizes are generally small, in eight out of ten profiles they lie above the lower bound of 0.96. It should also be noted that the data we used come from a population survey (Tromsø Study) and an online panel survey (MIC), thus we expect smaller magnitudes of differences than if using patient data. We observed similar results from the coefficient of determination (R-squared in Table 2): the highest total explained variance (*R*^*2*^) in EQ-VAS was 8.4% for the EQ-5D-5L profile (21231). In terms of Cohen’s f statistic, this is over 0.30, indicating a large effect size.

Heterogeneities by sex are reported in Appendix Table A5. For the two most prevalent profiles, results were similar in men and women. For the less prevalent profiles, results suggest stronger education gradients in women. In five of these, the gap between the highest and lowest education level was 5 points or more on the EQ-VAS. In other words, as compared to EQ-VAS, if EQ-5D-5L values are applied for measuring health inequalities in women, the social gradient would be more underestimated than when applied in men.

## Discussion

There is a growing interest in using EQ-5D as an outcome measure to evaluate health inequality. Different from previous literature that demonstrates the feasibility of using EQ-5D in studying health inequality [1–3, 5]. This study brings attention to researchers and policymakers to a critical observation that, as a health outcome, the EQ-5D could underestimate the SEP-health gradient, where SEP is proxied by educational attainment.

Based on data from a Norwegian population-based health study including more than 20,000 adults, and a multi-country MIC survey including more than 8,000 adults, our findings suggest that people who describe their health similarly on the EQ-5D-5L descriptive system differ systematically – depending on their educational attainment – in how they rate their health on the EQ-VAS. For the most prevalent profiles, we observed consistent gradients in EQ-VAS along each education level. However, in five less prevalent profiles, we observed a dip in mean EQ-VAS among subjects with the third highest education level (tertiary low) as compared to those with the second highest level (secondary school). Still, in nine out of ten EQ-5D-5L profiles considered, involving 80% of the total sample in the Norwegian data, the mean EQ-VAS was highest among individuals with the highest education level and lowest among those with the lowest education level. The multi-country MIC survey provided supportive evidence of this general pattern. Despite small effect sizes, the findings from the regression analyses also demonstrated consistent education gradients in EQ-VAS. The smaller effect sizes in the present study could be attributable to the relatively healthy samples identified according to the EQ-5D-5L classification system. Literature also suggests that MID varies based on baseline health and treatment types, where MID is higher with disease severity and lower baseline health [20].

We suggested two reasons to explain these systematic socioeconomic disparities in how individuals *describe* their health along the five dimensions included in the EQ-5D-5L descriptive system, as compared to how they *value* – or rate – ‘how good or bad their health is’ on a visual analogue scale. First, the role of *adaptation*; individuals with low socioeconomic position may have lower expectations and thereby *underreport* their health problems on a descriptive system [6]. The second reason is related to the omission of *psychosocial domains*, i.e. individuals with low socioeconomic position suffer more psychosocial problems [7], we tested this potential explanation by investigating variables in the two data sets that proxy important generic bolt-ons. When thinking about their health on a scale from ‘The best health you can imagine’ to ‘The worst health you can imagine’, individuals are likely to be aware of a wider range of dimensions *beyond* those covered by the EQ-5D. Thus, to the extent that the importance of these ‘hidden dimensions’ that respondents may have in their mind when answering the EQ-VAS differ across educational attainment, that may contribute to explaining the stronger EQ-VAS gradients. Recent studies have proposed the development of bolt-ons for the EQ-5D classification. The inclusion of such additional dimensions further increased the variation of EQ-VAS to be explained than using the original five dimensions alone [21, 22]. So far, there is no conclusion on which bolt-ons are most important for EQ-5D, and it is reasonable to expect that the answer would depend on the context EQ-5D is applied to, whether the general public or different patient groups.

In the Tromsø data, we considered three items to be aligned with the domains of *Social relationships* and *Community connectedness* [21, 23]. By merging these three items into a simple binary, good vs bad social relations, there was a consistent gradient that higher educational attainment had significantly higher odds for good social relations, e.g. individuals with the highest educational attainment had a 17.6% increased probability of having good social relationships compared to those with primary education (see Appendix Table A6). In the MIC data, we considered *vitality*, which has consistently been shown to play an important role across a wide range of respondent groups, such as people with chronic diseases, older adults, vulnerable people, and people who reported lower subjective SEP [21–24]. We found statistically significant, albeit weak, positive associations between education levels and vitality (Appendix Table A6). Hence, our explorative analyses suggest there are additional social gradients along these psychosocial domains in health, beyond those captured by the core dimensions of the EQ-5D, that partly explain the stronger educational gradients when HRQoL is measured on EQ-VAS. Further research is needed to bring more insights into which health dimensions have the strongest social gradients.

The magnitude of the observed disparities in EQ-VAS between the highest and lowest education levels has important implications for policy and practice. First, when national surveys seek to measure inequality in *quality of life*, we suggest supplementing the EQ-5D-5L descriptive system with psychosocial items, e.g. vitality and social relationships. If the aim is to monitor inequalities in *health*, we suggest including measures of chronic conditions or diagnoses in addition to subjective measures of HRQoL such as the EQ-5D and the EQ-VAS.

We acknowledge some limitations to this study. First, the empirical analyses to explore whether the EQ-5D-5L underestimates the education-health gradient were conducted against a single subjective outcome measure, EQ-VAS, of which the psychometric properties varied [25]. Future studies should consider using other health outcome measures to verify the robustness of the findings of this study [26, 27]. Second, the identified ten EQ-5D-5L profiles mainly refer to the relatively healthy respondents according to the EQ-5D-5L classification system. To verify the conclusion of this study among people in more severe health states, larger samples are required. Third, this study used educational attainment as a proxy for SEP. Other popular SEP indicators could also be used, such as income. However, the income variable is more likely to have reporting bias in the population survey, and it is more likely to be subject to reverse causality bias in the analysis. Fourth, the effect sizes cannot be compared across datasets because of differences in VAS wording and the number of education levels (4 in the Tromsø data, 3 in MIC). Lastly, we acknowledge the limited generalizability of the key findings, because all data come from high-income settings,

## Conclusion

Our results show that in the context of studying health inequalities, the important implication is that when measured by EQ-5D-5L values, the degree of inequality is lower than if measured by EQ-VAS scores. More research is needed to further investigate any additional gradients in psychosocial health domains that are not included in the EQ-5D, and the extent to which people with low socioeconomic position have lower expectations and thereby *underreport* their true health problems on a descriptive system. This calls for systematic integration of bolt-ons, and the use of mixed methods.

The implication of our results for policy and practice is to make planners aware that the population norms of EQ-5D-5L values tend to underestimate health inequalities. They should therefore include supplementary data on the social gradient in morbidity and mortality when targeting health inequalities.

## Supplementary Information

Below is the link to the electronic supplementary material.

Supplementary Material 3

## Data Availability

The Tromsø Study data are accessible upon approval. For more information, visit www.tromsostudy.com and www.tromsoundersokelsen.no. The MIC data is publicly available from the Centre for Health Economics, Monash University.
