# Supplementary material for: Measuring inequality in quality of life: Why the EQ-5D may underestimate it
Source: Qual Life Res. 2025 Nov 11;34(11):3283–90. doi: 10.1007/s11136-025-04087-7 (PMC12681450; doi:10.1007/s11136-025-04087-7)
Supplement: Supplementary file 1 — Supplementary Material 3 [file 11136_2025_4087_MOESM1_ESM.docx]

# Measuring inequality in quality of life: Why the EQ-5D may underestimate it

### Supplementary Material

**Appendix Table A1.** Frequency of low EQ-VAS scores for selected EQ-5D-5L profiles: Tromsø Study

| VAS score | Freq. | Percent | Cum. | VAS score | Freq. | Perc | Cum. | VAS score | Freq. | Perc | Cum. |
| --- | --- | --- | --- | --- | --- | --- | --- | --- | --- | --- | --- |
| ***11111*** |  |  |  | ***11122*** |  |  |  | ***11131*** |  |  |  |
| 5 | 2 | 0.04 | 0.04 | 3 | 1 | 0.07 | 0.07 | 5 | 2 | 0.30 | 0.30 |
| 6 | 1 | 0.02 | 0.05 | 10 | 1 | 0.07 | 0.14 | 10 | 1 | 0.15 | 0.45 |
| 7 | 1 | 0.02 | 0.07 | 15 | 2 | 0.14 | 0.28 | 20 | 1 | 0.15 | 0.60 |
| 8 | 1 | 0.02 | 0.09 | 25 | 2 | 0.14 | 0.42 | 25 | 2 | 0.30 | 0.90 |
| 10 | 1 | 0.02 | 0.11 | 30 | 9 | 0.63 | 1.04 | 27 | 1 | 0.15 | 1.05 |
| 20 | 3 | 0.05 | 0.16 | 35 | 4 | 0.28 | 1.32 | 30 | 4 | 0.60 | 1.65 |
| 25 | 1 | 0.02 | 0.18 | 40 | 13 | 0.9 | 2.22 | 35 | 1 | 0.15 | 1.80 |
| 30 | 4 | 0.07 | 0.25 | ***21121*** |  |  |  | 40 | 16 | 2.39 | 4.19 |
| 33 | 2 | 0.04 | 0.28 | 10 | 1 | 0.15 | 0.15 | ***11132*** |  |  |  |
| 35 | 1 | 0.02 | 0.30 | 20 | 2 | 0.31 | 0.46 | 20 | 3 | 1.28 | 1.28 |
| 40 | 13 | 0.23 | 0.53 | 25 | 2 | 0.31 | 0.77 | 25 | 1 | 0.43 | 1.71 |
| 45 | 5 | 0.09 | 0.61 | 30 | 6 | 0.93 | 1.70 | 30 | 1 | 0.43 | 2.14 |
| 50 | 127 | 2.29 | 2.92 | 35 | 1 | 0.15 | 1.85 | 35 | 2 | 0.85 | 2.99 |
| ***11121*** |  |  |  | 40 | 8 | 1.24 | 3.09 | 40 | 2 | 0.85 | 3.85 |
| 0 | 1 | 0.02 | 0.02 | ***11112*** |  |  |  | ***21231*** |  |  |  |
| 5 | 2 | 0.03 | 0.05 | 0 | 1 | 0.18 | 0.18 | 30 | 4 | 2.03 | 2.03 |
| 8 | 2 | 0.03 | 0.09 | 10 | 1 | 0.18 | 0.36 | 35 | 1 | 0.51 | 2.54 |
| 9 | 2 | 0.03 | 0.12 | 30 | 3 | 0.53 | 0.89 | 40 | 8 | 4.06 | 6.60 |
| 10 | 2 | 0.03 | 0.15 | 35 | 2 | 0.35 | 1.24 | 45 | 14 | 3.77 | 10.37 |
| 15 | 1 | 0.02 | 0.17 | 40 | 4 | 0.71 | 1.95 | 50 | 42 | 21.32 | 31.69 |
| 20 | 6 | 0.10 | 0.27 | ***11221*** |  |  |  | ***11123*** |  |  |  |
| 25 | 8 | 0.14 | 0.41 | 4 | 1 | 0.24 | 0.24 | 25 | 1 | 0.52 | 0.52 |
| 30 | 10 | 0.17 | 0.58 | 25 | 1 | 0.24 | 0.49 | 35 | 2 | 1.05 | 1.57 |
| 35 | 5 | 0.09 | 0.67 | 30 | 1 | 0.24 | 0.73 | 40 | 5 | 2.62 | 4.19 |
| 40 | 27 | 0.47 | 1.14 | 35 | 2 | 0.49 | 1.22 | 45 | 5 | 2.62 | 6.81 |
| 42 | 1 | 0.02 | 1.16 | 40 | 4 | 0.97 | 2.19 | 50 | 18 | 9.42 | 16.23 |

*EQ-VAS*: EuroQol Visual Analogue Scale. Investigated for reasons of exclusion due to suspected inconsistent responses between EQ-VAS and EQ-5D-5L, where respondents with EQ-VAS scores less than 50 for profile 11111 (close to 3 SD variation) and less than 30 for all other profiles were excluded. The highlighted frequencies indicate *excluded samples.*

**Appendix Table A2.** Description of the most frequent profiles, and the most frequent profiles that include at least one dimension described at level 3, 4 or 5.

|  | EQ-5D-5L | | EQ-VAS | | | Excluded cases* |
| --- | --- | --- | --- | --- | --- | --- |
| Profile | | Index value | N (%) | Cum. Freq. (%) | Mean (SD) |  |
| **Tromsø Study (40-79 years)** | |  |  |  |  |  |
| *Most frequent profiles* | |  |  |  |  |  |
| 11111 | | 1.000 | 5,557 (28.5) | 28.5 | 84.3 (11.8) | 35 |
| 11121 | | 0.930 | 5,778 (29.6) | 58.2 | 80.3 (12.4) | 24 |
| 11122 | | 0.869 | 1439 (7.4) | 65.5 | 76.2 (13.4) | 6 |
| 11131 | | 0.894 | 670 (3.4) | 69.0 | 71.1 (15.1) | 7 |
| 21121 | | 0.872 | 647 (3.3) | 72.3 | 75.5 (14.4) | 5 |
| 11112 | | 0.929 | 567 (2.9) | 75.2 | 78.6 (13.6) | 2 |
| 11221 | | 0.883 | 411 (2.1) | 77.3 | 72.5 (14.1) | 2 |
| *Moderate level profiles* | |  |  |  |  |  |
| 11132 | | 0.833 | 234 (1.2) | 78.5 | 67.4 (14.4) | 4 |
| 21231 | | 0.789 | 197 (1.0) | 79.5 | 62.0 (13.8) | 0 |
| 11123 | | 0.807 | 191 (1.0) | 80.5 | 69.1 (13.5) | 1 |
| **MIC data (25-79 years)** | |  |  |  |  |  |
| *Most frequent profiles* | |  |  |  |  |  |
| 11111 | | 1.000 | 1,365 (18.5) | 18.5 | 82.8 (12.0) | 26 |
| 11121 | | 0.930 | 1,079 (14.7) | 33.2 | 78.7 (13.5) | 8 |
| 11122 | | 0.869 | 617 (8.4) | 41.6 | 72.6 (14.8) | 6 |
| 11112 | | 0.929 | 365 (5.0) | 46.6 | 76.6 (14.6) | 2 |

*EQ-5D-5L* with mean values from a hybrid (*MN-WePP*) based on 10 Western countries’ value sets; *EQ-VAS*: EuroQol Visual Analogue Scale; *MIC*: Multi-Instrument Comparison study; *SD*: Standard Deviation. *Respondents with VAS scores less than 50 for profile 11111 (close to 3 SD variation) and less than 30 for all other profiles were excluded.

**Appendix Table A3.** Linear regression of EQ-VAS score on educational attainment within the three most prevalent EQ-5D-5L profiles, adjusted for age and country dummies: MIC data (Age 25-79 years)

|  | Variation in VAS within identical EQ-5D-5L profiles | | |  |
| --- | --- | --- | --- | --- |
| Variables | 11111 | 11121 | 11122 | 11112 |
| Education |  |  |  |  |
| Diploma | 0.863 | 0.974 | 0.849 | -0.143 |
|  | (0.690) | (0.940) | (1.360) | (1.883) |
| University | 2.001*** | 1.476 | 3.082** | 1.626 |
|  | (0.683) | (0.962) | (1.380) | (1.703) |
| Sex |  |  |  |  |
| Female | 1.251** | 1.862** | 1.342 | 2.127 |
|  | (0.536) | (0.723) | (1.061) | (1.392) |
| Chronic conditions |  |  |  |  |
| Diagnosed | -6.282*** | -8.006*** | -10.558*** | -9.253*** |
|  | (0.543) | (0.660) | (0.951) | (1.268) |
|  |  |  |  |  |
| Constant | 81.354*** | 80.345*** | 75.431*** | 81.219*** |
|  | (1.329) | (2.073) | (2.398) | (3.028) |
|  |  |  |  |  |
| Observations | 1 339 | 1 071 | 611 | 363 |
| R-squared | 0.104 | 0.108 | 0.157 | 0.134 |

*EQ-VAS*: EuroQol Visual Analogue Scale. Robust standard errors are in parentheses.

*** p<0.01, ** p<0.05, * p<0.1.

**Appendix Table A4.** Fractional logit regressions of EQ-VAS score on education attainment within selected EQ-5D-5L profiles: The Tromsø Study (40-79 years)

|  | Most prevalent profiles | | | | | | Moderate level profiles | | | |
| --- | --- | --- | --- | --- | --- | --- | --- | --- | --- | --- |
| Variables | 11111 | 11121 | 11122 | 21121 | 11112 | 11221 | 11131 | 11132 | 21231 | 11123 |
| Education |  |  |  |  |  |  |  |  |  |  |
| Secondary | 0.940* | 1.6924*** | 0.208 | 2.219 | 3.098 | 2.052 | 3.045** | 1.629 | 5.802** | 0.855 |
|  | (0.514) | (0.504) | (1.032) | (1.472) | (1.919) | (1.804) | (1.445) | (2.305) | (2.429) | (2.982) |
| Tertiary low | 1.728*** | 2.5889*** | 0.465 | 1.268 | 2.179 | 2.374 | 2.004 | -1.170 | 6.548* | 0.6451 |
|  | (0.518) | (0.516) | (1.142) | (1.800) | (1.966) | (2.110) | (1.807) | (2.577) | (3.421) | (3.280) |
| Tertiary high | 2.452*** | 3.712*** | 1.731* | 5.733*** | 4.169** | 3.752* | 2.928* | 2.354 | 10.871*** | 4.979 |
|  | (0.477) | (0.478) | (1.002) | (1.487) | (1.872) | (2.051) | (1.523) | (2.532) | (2.813) | (3.088) |
| Sex |  |  |  |  |  |  |  |  |  |  |
| Female | 2.7655*** | 1.961*** | 1.312* | 0.802 | 1.435 | -1.479 | 0.847 | 0.158 | -0.009 | -0.078 |
|  | (0.298) | (0.308) | (0.690) | (1.088) | (1.096) | (1.404) | (0.01.1107) | (1.715) | (2.248) | (1.869) |
|  |  |  |  |  |  |  |  |  |  |  |
| Observations | 5,440 | 5,695 | 1,417 | 631 | 556 | 407 | 653 | 230 | 195 | 189 |
| R-squared | 0.023 | 0.022 | 0.007 | 0.033 | 0.014 | 0.014 | 0.021 | 0.013 | 0.083 | 0.024 |

Standard errors in parentheses

*** p<0.01, ** p<0.05, * p<0.1

**Appendix Table A5.** Linear regression of EQ-VAS score on educational attainment ***by sex***: Tromsø data (Age 40-79 years)

|  | Most prevalent profiles | | | | | | Moderate level profiles | | | |
| --- | --- | --- | --- | --- | --- | --- | --- | --- | --- | --- |
| Variables | 11111 | 11121 | 11122 | 21121 | 11112 | 11221 | 111**3**1 | 111**3**2 | 212**3**1 | 1112**3** |
|  | ***Male*** |  |  |  |  |  |  |  |  |  |
| Education |  |  |  |  |  |  |  |  |  |  |
| Secondary | 1.112* | 1.176* | -1.857 | 2.060 | 0.682 | -0.171 | 2.570 | 2.895 | 2.027 | 1.203 |
|  | (0.673) | (0.686) | (1.451) | (2.033) | (2.738) | (2.571) | (1.957) | (3.478) | (5.324) | (3.905) |
| Tertiary low | 1.596** | 1.813** | -2.718 | 0.771 | 0.999 | -2.002 | -1.901 | -1.565 | 8.174 | 2.534 |
|  | (0.676) | (0.713) | (1.661) | (2.192) | (2.778) | (3.307) | (2.433) | (4.122) | (6.635) | (4.050) |
| Tertiary high | 2.814*** | 3.899*** | 0.164 | 4.522** | 2.623 | 2.135 | 0.442 | 5.321 | 7.762 | 5.339 |
|  | (0.642) | (0.664) | (1.389) | (2.061) | (2.698) | (3.741) | (2.252) | (3.371) | (7.228) | (4.430) |
|  |  |  |  |  |  |  |  |  |  |  |
| Constant | 79.848*** | 77.010*** | 76.068*** | 78.102*** | 74.022*** | 82.791*** | 77.533*** | 70.183*** | 51.105*** | 64.070*** |
|  | (1.344) | (1.409) | (3.265) | (4.681) | (5.494) | (6.693) | (4.834) | (7.004) | (11.650) | (8.509) |
|  |  |  |  |  |  |  |  |  |  |  |
| Observations | 2,947 | 2,785 | 590 | 322 | 290 | 152 | 307 | 96 | 52 | 101 |
| R-squared | 0.008 | 0.014 | 0.009 | 0.023 | 0.006 | 0.024 | 0.020 | 0.047 | 0.057 | 0.023 |
|  | ***Female*** |  |  |  |  |  |  |  |  |  |
| Education |  |  |  |  |  |  |  |  |  |  |
| Secondary | 0.657 | 2.190*** | 2.013 | 2.289 | 5.470** | 4.210* | 3.549 | 0.793 | 6.790** | 0.215 |
|  | (0.785) | (0.746) | (1.481) | (2.149) | (2.600) | (2.540) | (2.159) | (3.239) | (2.804) | (4.614) |
| Tertiary low | 1.922** | 3.246*** | 2.977* | 1.809 | 2.654 | 5.704** | 6.350** | -0.506 | 3.281 | -2.442 |
|  | (0.801) | (0.751) | (1.594) | (3.224) | (2.802) | (2.779) | (2.671) | (3.487) | (3.466) | (5.833) |
| Tertiary high | 2.016*** | 3.458*** | 3.148** | 6.808*** | 5.235** | 5.558** | 5.007** | 0.352 | 11.357*** | 4.238 |
|  | (0.702) | (0.694) | (1.462) | (2.156) | (2.600) | (2.586) | (2.131) | (3.776) | (3.005) | (4.517) |
|  |  |  |  |  |  |  |  |  |  |  |
| Constant | 83.802*** | 81.895*** | 77.510*** | 76.987*** | 71.241*** | 65.782*** | 74.752*** | 72.055*** | 68.026*** | 68.738*** |
|  | (1.582) | (1.559) | (3.244) | (5.344) | (6.608) | (5.737) | (5.476) | (8.624) | (7.444) | (11.434) |
|  |  |  |  |  |  |  |  |  |  |  |
| Observations | 2,493 | 2,910 | 827 | 309 | 266 | 255 | 346 | 134 | 143 | 88 |
| R-squared | 0.005 | 0.018 | 0.012 | 0.038 | 0.025 | 0.025 | 0.038 | 0.004 | 0.127 | 0.033 |

*EQ-VAS*: EuroQol Visual Analogue Scale. Robust standard errors in parentheses, *** p<0.01, ** p<0.05, * p<0.1. All models controlled for age.

**Appendix Table A6.** Regression of two psycho-social domains on educational attainments

|  | Tromsø study | MIC study^A^ |
| --- | --- | --- |
| Variables | Social relationships^B^ | Vitality^C^ |
| Education |  |  |
| Secondary | 0.086*** | 0.528 |
|  | (0.013) | (0.735) |
| Tertiary low | 0.132*** | 1.561** |
|  | (0.014) | (0.749) |
| Tertiary high | 0.176*** |  |
|  | (0.013) |  |
| Constant | 0.283*** | 62.098*** |
|  | (0.030) | (1.548) |
|  |  |  |
| Observations | 12 576 | 3 061 |
| R-squared | 0.028 | 0.237 |

Standard errors in parentheses, *** p<0.01, ** p<0.05, * p<0.1

^A^ Educational attainment in the MIC survey was split into three categories (High school, Diploma/certificate, University). Analyses are based on pooled cases from the three most prevalent 5L profiles (11111, 11121, 11122). All results are adjusted for age, sex, and dummies for the three most prevalent 5L profiles. For the MIC study, we further adjusted for chronic conditions and country dummies.

^B^ Social relationship was captured by three items: i) participate in organized gatherings (Never or a few times a year vs 1–3 times a month or more frequent); ii) have friends to talk with (No vs Yes); iii) have friends who give support (No *vs.* Yes). Respondents who answered *Yes* to all three questions were classified as having good social relationships, and the rest were classified as *not* having good social relationships, i.e., social relationship is a binary variable. For easy interpretation, we applied linear probability modelling to estimate the association between education and social relationships. For instance, respondents with secondary education had an 8.6 per cent increased probability of having good social relationships compared to those with primary education.

^C^ Vitality was measured by four items from the 36-Item Short-Form Survey (SF-36): i) Did you feel full of life? (item 23); ii) Did you have a lot of energy? (item 27); iii) Did you feel worn out? (item 29), and; iv) Did you feel tired? (item 31). Each item has five response choices: *All of the time*, *Most of the time*, *Some of the time*, *A little of the time*, and *None of the time*. The first two questions are reverse-coded so that a high score defines a more favourable health state. Then, each item is scored on a 0 to 100 range so that the lowest and highest possible scores are 0 and 100, respectively. Scores represent the percentage of the total possible score achieved. Finally, the scores of the four items are averaged together to create the vitality scale score, ranging from 0 to 100. A linear regression model has been applied to estimate the effect of education on vitality/energy.


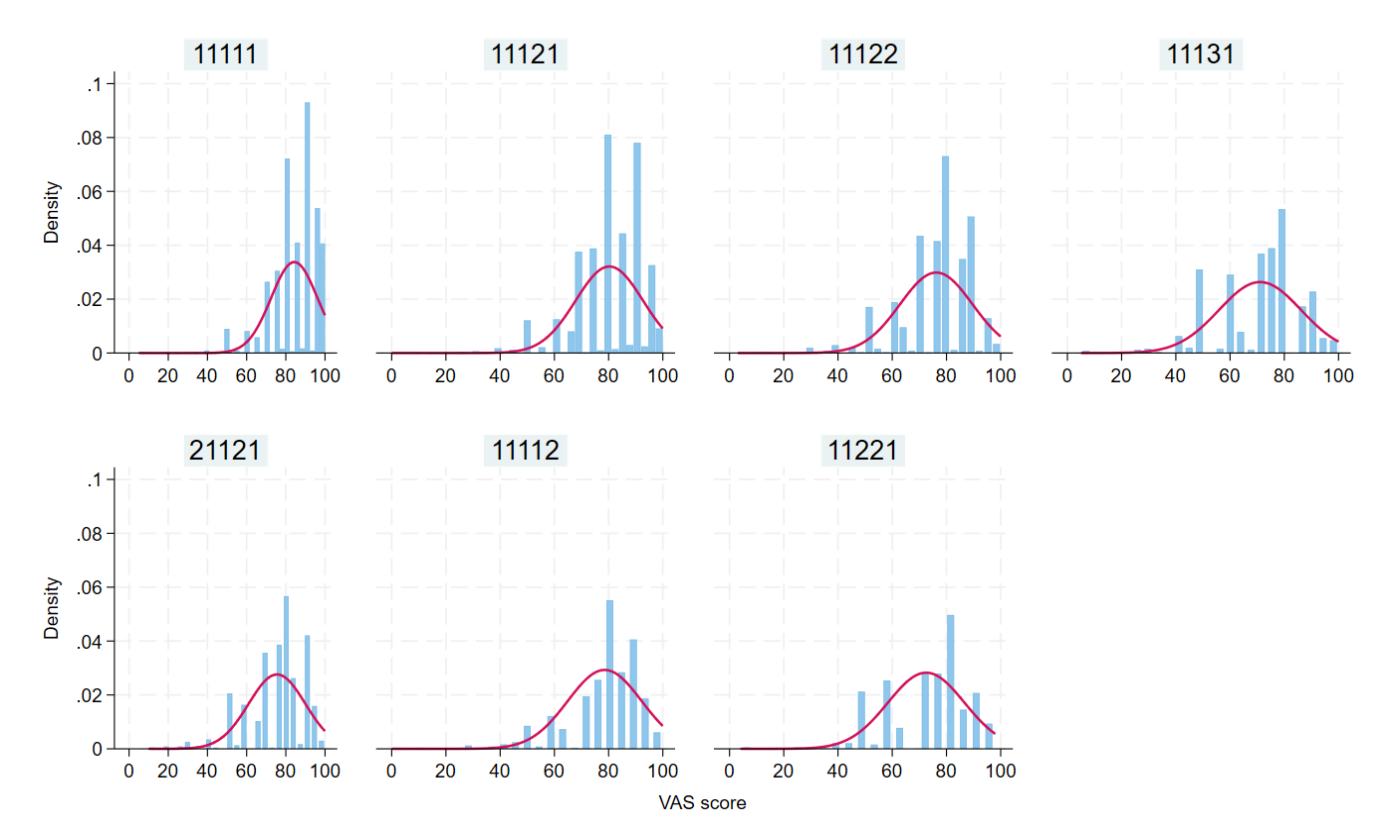


*EQ-VAS:* EuroQoL Visual Analogue Scale.

**Appendix Figure A1.** The distribution of EQ-VAS for the most prevalent EQ-5D-5L profiles: The Tromsø Study (Age 40-79 years)


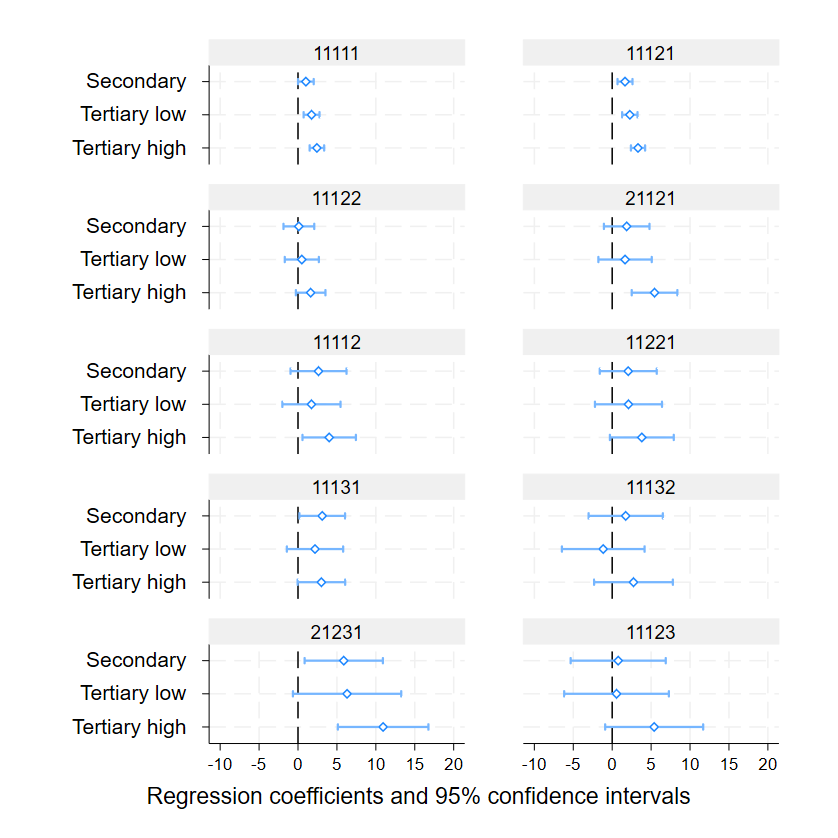


**Appendix Figure A2.** Plots of regression coefficients for ten EQ-5D-5L profiles; robust regression, The Tromsø Study (Age 40-79 years)

*EQ-VAS:* EuroQol Visual Analogue Scale.
